# Development of the Dissemination and Implementation Science Collaborative (DISC): Opportunities to enhance implementation science capacity among researchers and practitioners in South Carolina

**DOI:** 10.1017/cts.2024.579

**Published:** 2024-10-14

**Authors:** Caitlin G. Allen, Katherine Sterba, Stephanie Oppenheimer, Rochelle F. Hanson, Emma Coen, Ron Gimbel, Dee Ford

**Affiliations:** 1 Medical University of South Carolina, Charleston, SC, USA; 2 Clemson University, Clemson, SC, USA

**Keywords:** Capacity building, translational research, dissemination and implementation science

## Abstract

This paper explores the development of the Dissemination and Implementation Science Collaborative (DISC) at the Medical University of South Carolina, established through the Clinical and Translational Science Award program. DISC aims to accelerate clinical and translational science by providing training, mentorship, and collaboration opportunities in dissemination and implementation (D&I) science. Through DISC, investigators, trainees, and community partners are equipped with the knowledge and skills to conduct D&I research and translate findings into practice, particularly in South Carolina’s public health and healthcare landscape. We describe efforts to achieve the major overarching aims of DISC, which include conducting scientific workforce training, providing mentorship and consultation, and advancing methods and processes for D&I research. By sharing DISC experiences, successes, and challenges, this paper aims to support the growth of D&I research and capacity-building programs, fostering collaboration and shared resources in the field.

## Introduction

Dissemination and implementation (D&I) science has rapidly come to prominence as a field in the past two decades [1–3]. Dissemination studies focus on the targeted distribution of information and materials to advance the spread of interventions and innovation. Relatedly, implementation studies examine methods to promote the integration of evidence-based innovations, interventions, and policies into practice. Taken together, D&I research aims to accelerate the timely translation of evidence-based practice and policy by designing studies to better understand how interventions, practices, and innovations are launched and executed in specific settings [4,5].

Interest in D&I science has grown exponentially and can, in part, be attributed to increased investment and attention from federal funding agencies including the National Institutes of Health (NIH), Fogarty International Center, Patient Centered Outcomes Research Institute, and the Clinical and Translational Science Award (CTSA). For example, the NIH, one of the largest funders of D&I research, has a robust program of funding announcements, spearheaded training programs, an annual meeting, and standing study section to advance the field [6]. Concurrently, research centers and programs focused on D&I training, mentorship, and capacity building have also proliferated, with support from these funding agencies, to meet the growing interest and demand for D&I scientists. A recent review catalogued 165 D&I programs with a range of program goals, including capacity building through targeted consultations, providing technical assistance, and development of toolkits to support the application of D&I science in research and clinical settings [7]. This growing emphasis on D&I research has been sparked by recognition of the value of D&I science in improving, scalability, and sustainability of interventions, as well as the need to proactively integrate D&I research to ensure federally funded research is fully integrated into practice for population-level improvements.

Through our institution’s CTSA program, the Medical University of South Carolina (MUSC) has established the Dissemination and Implementation Science Collaborative (DISC) as part of the South Carolina Translational Research Institute (SCTR). The CTSA currently funds 64 institutions and 156 partner sites across the USA. As part of the current CTSA program, sites are required to embed principles of D&I science throughout their respective programs, including multidisciplinary capacity-building services and collaboration with other CTSA sites to create solutions to implement evidence into practice in different academic medical centers [8]. The DISC program focuses on accelerating clinical and translational research in later stages of the translational spectrum to support the implementation of research into practice within our institution’s learning health system at MUSC and across the state of South Carolina.

DISC was established through the CTSA in 2019 and strategically leverages infrastructure and funding resources within SCTR to provide opportunities for investigators, trainees, and community partners to use implementation science principles and conduct D&I research to accelerate the impact of clinical and translational research on health and healthcare needs in priority areas in South Carolina. The three guiding aims of DISC are to (1) conduct scientific workforce training in D&I science to increase knowledge, capacity, and skills in the design and conduct of D&I research; (2) provide mentorship, consultation, and collaboration to investigators in developing D&I research; and (3) advance methods and processes for D&I by developing a series of clinical platforms that lower barriers to the conduct of D&I research and accelerate clinical and translational research.

As investments in D&I science continue to grow, sharing approaches and best practices to support burgeoning D&I research and capacity-building programs will support opportunities for shared resources, collaboration, and growth within the field. The goals of this paper are to describe DISC efforts across each of our site’s aims and share successes, lessons learned, and future directions. Sharing experiences can help as new D&I centers are established.

## Activities and accomplishments

### Overview of DISC and guiding framework

Established in 2009 through the CTSA award, SCTR aims to transform the biomedical research culture in South Carolina by facilitating resource sharing and streamlining research processes. The overarching goal is to “be the catalyst for changing the culture of biomedical research in South Carolina… facilitate the sharing of resources and expertise and streamline research-related processes to bring about large-scale change in the [South Carolina]’s clinical and translational research efforts.” DISC was established through the CTSA infrastructure in 2019 to enhance the state’s research culture by emphasizing the value of D&I science by offering shared resources and support. Both SCTR and DISC focus on rural and underserved populations, investing in infrastructure for D&I science at MUSC. DISC utilizes the SCTR website and an online platform (SPARCRequest) for content, resources, and service requests. An 11-member internal advisory panel, which includes faculty from MUSC with expertise in telehealth, clinical implementation, health services research, and the learning health system, has worked with DISC leadership to provide feedback about the direction of the program, focusing on workforce training, mentorship, and advancing D&I methods.

### Aim 1: Conduct scientific workforce training in D&I science to increase knowledge, capacity, and skills in the design and conduct of D&I research

#### D&I science needs assessment

We developed a needs assessment to help characterize current D&I science occuring at our institution and identify interest in D&I research training and support needs. As part of the survey, we defined implementation science as “the study of methods to promote the integration of research findings into healthcare policy and practice,” dissemination research as, “studies focused on the targeted distribution of information and materials to advance the spread of evidence about interventions and innovations,” and implementation research as “studies focused on understanding implementation processes and outcomes and identifying effective strategies for integrating new evidence-based practices/innovations within a specific setting.”

The survey was distributed in October 2021 through SCTR’s newsletters and targeted department and workgroup listservs, as well as personal networks, with a focus on capturing needs of investigators interested in D&I science. The survey included questions about the individual’s institutional affiliation, research interests, clinical focus, and D&I experience and funding. Guided by our team’s experience with consultations and literature examining barriers and training needs in D&I [9,10], we captured key barriers to conducting D&I research by including pre-identified potential challenges (time, lack of support staff, finding collaborators, funding opportunities, regulatory issues regarding D&I methods, lack of access to training, lack of access to a mentor, difficulty breaking into the field), which were ranked on a Likert scale (not a barrier at all, minor barrier, moderate barrier, major barrier). Individuals were then asked to rank their knowledge of a topic (low = 1 to high = 5) and their interest in receiving training on this topic (low = 1 to high = 5) across a range of domains: general principles of D&I research, funding opportunities, grant writing, institutional review board (IRB) applications, frameworks, study design, measurement, mixed methods, clinical guidance, intervention adaptation, digital health and technology, policy change, and health equity. Respondents also were asked to rank their interest in a range of services (Likert scale, 1=not at all interested to 5=extremely interested), including one-on-one consultations, lectures from content experts in the field, participation in research group meetings, journal club, retreats to share research and network, lectures and training for professional educational credit, full semester courses, and multi-session workshops.

Among those who reported their current role (*n* = 44), the majority (*n* = 33, 75%) indicated they were researchers, followed by clinicians/researchers (*n* = 7, 15.9%), educators (*n* = 5, 11.4%) and clinicians (*n* = 4, 9.1%). Most individuals reported having conducted D&I research in the past (*n* = 36/44, 67.9%), with an average of 6.71 years conducting D&I research (StDev = 6.3) and rated themselves as “intermediate” (*n* = 15/36, 41.7%) in their expertise in conducting D&I research.

The top-rated barrier to conducting D&I research was “finding time” (*M* = 2.9/4, SD = 0.9), followed by “difficult to break into the field” (*M* = 2.8/4, SD = 0.9) and “lack of support staff” (*M* = 2.6/4, SD = 0.9). (Table 1).

**Table 1.** Barriers and requested support services (*n* = 50)

	Mean score	Standard deviation
Barrier to conducting D&I research (1–4 Likert scale: 1 not a barrier at all, 4 major barriers)		
Regulatory issues regarding D&I methods	2	0.9
Lack of access to a mentor	2.2	0.9
Lack of access to training opportunities (e.g., seminars, short courses)	2.3	1.0
Finding collaborators to serve on the study	2.4	1.0
Finding funding opportunities	2.6	1.2
Lack of support staff	2.6	0.9
Difficult to break into the field	2.8	0.9
Finding time	2.9	0.9
Rate your interest in the following specific D&I support services (1–5)		
Lectures and trainings for professional educational credit	2.6	1.4
Journal club	2.8	1.2
One semester – weekly d&i sessions	2.9	1.3
Retreats to share research and network	3.2	1.2
Multi-session workshop	3.4	1.1
One-on-one consultations	3.6	1.2
Lectures from content experts in the field	3.6	1.2
Participation in research group meetings	3.6	1.2

D&I = dissemination and implementation.

Individuals rated “IRB applications” (*M* = 3.4/5, SD = 1.4), “mixed methods” (*M* = 3.2/5, SD = 1.3), and “grant writing” (*M* = 3.1/5, SD = 1.3) as being their highest areas of knowledge in the D&I area (Table 2). The lowest areas of knowledge included “funding opportunities” (*M* = 2.7/5, SD = 1.1), “clinical guidelines,” (*M* = 2.4/5, SD = 1.3), and “policy change” (*M* = 2.3/5, SD = 0.9). Top-rated training interest areas included “intervention adaptation” (*M* = 3.9/5, *M* = 1.2,), “study design” (*M* = 3.9/5, SD = 1.2), “general principles of D&I research” (*M* = 3.9/5, SD = 1.2), and “frameworks” (*M* = 3.9/5, SD = 1.3). Top-rated support services included participation in research group meetings (*M* = 3.6/5, SD = 1.2), lectures from content experts in the field (*M* = 3.6/5, SD = 1.2), and one-on-one consultations (*M* = 3.6/5, *M* = 1.2). The average maximum number of hours an individual planned to devote to D&I training was 30.6 (SD = 31.5) over the next year.

**Table 2.** Knowledge gaps and training interests

	Knowledge		Training interest	
	Mean	Standard deviation	Mean	Standard deviation
IRB applications	3.4	1.4	2.6	1.4
Mixed methods	3.2	1.3	3.3	1.1
General principles of D&I research	3.0	1.2	3.9	1.2
Measurement	3.0	1.3	3.7	1.3
Grant writing	3.1	1.3	3.5	1.3
D&I health equity topics	2.9	1.1	3.7	1.3
Digital health/technology	2.9	1.2	3.6	1.2
Study design	2.8	1.1	3.9	1.2
Intervention adaptation	2.8	1.0	3.9	1.2
Frameworks	2.8	1.2	3.9	1.3
Funding opportunities	2.7	1.1	3.7	1.4
Clinical guidelines	2.4	1.2	3.6	1.4
Policy Change	2.3	0.9	3.1	1.2

Likert Scale: 1–5 (low = 1 to high = 5).

D&I = dissemination and implementation; IRB = institutional review board.

#### D&I science retreat capacity-building efforts

Informed by a needs assessment and advisory committee input, we co-hosted a scientific retreat with SCTR in September 2022. The retreat catered to varying levels of implementation science experience and clinical focus, featuring presentations and workshops led by faculty and students from Clemson, USC, and MUSC, including a keynote by Dr. Mark McGovern from Stanford University. DISC faculty co-led workshops. Post-retreat, we conducted an evaluation in October 2022 to gauge attendee satisfaction and impact. Of the respondents, the majority were faculty (23/47, 54.8%), staff (6/42, 14.3%), and students (4/42, 9.5%). Additional participants included post-doctoral fellows (*n* = 3, 7.1%), Other (*n* = 3, 7.1%), community members (*n* = 2, 4.8%), and clinicians (*n* = 1, 2.4%). The majority of respondents (29/42, 69%) were engaged in D&I research. Overall, participants highly recommended the retreat (*M* = 8.9) with some differences in likelihood to recommend based on type of participant. Community members (*n* = 2) were least likely to recommend the retreat (*M* = 7.5/10) and students were most likely to recommend the retreat (*M* = 9.5/10). The keynote and oral presentations were rated most helpful, with “D&I Conceptual Frameworks” as the standout breakout session. The session covered the importance of theories, models, and frameworks (TMFs), offering insights and case studies on TMF selection. The majority of participants strongly agreeing they learned about new resources (*M* = 4.4/5), with some differences in agreement with the amount they learned across types of attendees. Staff were least likely to indicate they learned about new resources (*M* = 3.4/5) and students were most likely to indicate that they learned about new resources (*M* = 4.8/5). Respondents indicated that they intend to integrate retreat insights within six months (*M* = 4.3/5).

#### Ongoing training and resources

DISC supports D&I capacity building through the retreat as well as ongoing activities such as D&I interest group sessions, a web-based resource library, and participation in regional and national training initiatives. The interest group hosts monthly sessions covering various implementation science topics, while the regularly updated DISC website provides resources for independent learning, including educational opportunities, research retreats, and funding announcements. Thematic organization includes guidance on D&I models, study designs, and measurement tools. The team also shares insights through an “ask the experts” panel. Additionally, DISC engages in cross-CTSA collaborations and national D&I efforts through workforce trainings hosted by societies (e.g., National Society of Genetic Counseling symposium on implementation science), other CTSAs (e.g., University of Florida CTSA), and participation in various training activities (e.g., journal clubs, HRSA Practice Transformation and Population Health Fellowship quarterly seminar series).

### Aim 2: Provide mentorship, consultation, and collaboration to investigators in developing D&I research

DISC uses an online request center to track mentorship, consultations, and collaborations. This web-based system streamlines research service requests, pricing, and application submissions with a focus on billing compliance and proposal development. DISC faculty provides consultations for crafting robust D&I sections in proposals, offering concept review, designing theory-based interventions, and facilitating multidisciplinary collaboration. To date, DISC has supported 28 grant submissions with five successfully funded. Additionally, DISC collaborates with MUSC Health’s Value Institute to contribute to the learning health system’s emphasis on quality through consultative support and consistency in D&I science adoption. DISC members also lead cross-CTSA collaborative research to advance D&I frameworks, measurement tools, and best practices, fostering connections with other institutions and contributing to the CTSA’s Dissemination, Implementation, and Knowledge Transfer working group.

### Aim 3: Advance methods and processes for D&I by developing a series of clinical telehealth platforms that lower barriers to the conduct of D&I research and accelerate clinical and translational research

We have advanced D&I research methods by integrating principles into telehealth program evaluation and subsequently developing a dynamic toolkit for interprofessional telehealth teams grounded in lessons learned. Initially, DISC applied D&I methods to three telehealth programs as part of their evaluation. Experience from this process informed the toolkit. Toolkit development involved a literature review, content framing, interdisciplinary team feedback, and adhering to AHRQ toolkit checklist standards. Following internal review (*n* = 5) and external evaluation (*n* = 3) for acceptability and usability, the toolkit is now accessible on the DISC website. Presently, the toolkit is being applied prospectively in telehealth evaluation and is adaptable for various settings.

DISC supported research projects with the MUSC Center of Excellence in Telehealth to advance the science of D&I at MUSC. We designed and completed three studies in collaboration with MUSC telehealth teams examining barriers and facilitators to telehealth program delivery from the perspectives of diverse stakeholders. First, we examined school-based telehealth asthma care in South Carolina using focus groups (*n* = 11) and surveys with school nurses, teachers, and administrators (*n* = 34) [11–13]. Next, we completed a study evaluating determinants of implementation outcomes in a statewide remote patient monitoring program for diabetes and hypertension. We completed focus groups (*n* = 10) and administered champion surveys in primary care (including free and FQHC) clinics [14]. Finally, we completed six focus groups with OB-Gyn and pediatric clinics and administered site surveys and patient surveys to evaluate the implementation of the Women’s Reproductive & Behavioral Health program, a maternal mental health and substance use disorder telehealth program for pregnant women [15,16].

Other telehealth implementation efforts have included support for intensive care unit innovations as a tool for the implementation of mechanical ventilation best practices in rural settings. Through this collaboration, DISC used the EPIS framework to develop tools (e.g., site surveys, staff readiness assessments, implementation tracking log, and focus group guide) to monitor the implementation processes and outcomes. We identified key program delivery factors (e.g., collaborative engagement style, communication) and stakeholder factors (e.g., leadership, staff attitudes, teamwork) driving uptake and fidelity [17,18]. The result of this work guided the development of a digital/web-based program that provides asynchronous multidisciplinary learning, followed by interactive virtual facilitation between partner intensive care teams and the MUSC intensive care unit’s innovations’ team.

DISC also actively integrates D&I into clinical research in high-need rural communities served by MUSC, such as the Clemson-MUSC Health Extension Program, featuring telehealth-equipped mobile clinics. Additionally, DISC collaborates with the MOVENUP program, employing a “train the trainer” approach to inform community health educators about prevention, screening, and treatment options, including clinical trials. Leading implementation science and evaluation for a population-wide genomic screening program across MUSC, DISC assesses multilevel factors influencing the successful implementation of the In Our DNA SC initiative [19]. This includes developing data collection tools like surveys, interview guides, and tracking logs to capture and code work group meetings, adaptations, and technical assistance needs [20].

## Discussion

The D&I science efforts at MUSC have been growing since the establishment of DISC through the CTSA in 2019. DISC has successfully leveraged MUSC’s infrastructure and resources to advance opportunities for researchers, trainees, and community partners by conducting scientific workforce trainings about D&I science, providing mentorship and consultation to support developing of D&I research, and establishing innovative platforms that lower the barriers to the conduct of D&I research. Through this article, we sought to share experiences and lessons learned in the ongoing D&I initiatives at MUSC.

### Workforce development and training

A major responsibility for DISC is capacity building and workforce development to support D&I research. In our needs assessment, we found that most respondents indicated that they previously conducted D&I work and less than half considered themselves to be “intermediate” in D&I skills. These findings align with other national surveys of CTSA leadership that show many investigators are at least familiar with D&I [21]. Despite familiarity with D&I, our experience, as well as those reported elsewhere, indicates a strong emphasis on supporting trainings, providing consultations, and workforce development for those interested in D&I methods [22]. A national CTSA survey indicated that substantial time is spent across CTSAs providing consultations and mentorship programs, as well as workshops and seminars, with workforce development commonly identified as a key priority [23]. Indeed, the type of services most requested by our researchers included: research meetings, lectures, and one-on-one consultations, with respondents indicating they would dedicate substantial time (30 hours) to additional training in D&I. Nationally, the majority (70.3%) of CTSAs are funding training and workforce development either directly through the CTSA or indirectly, with the goal of expanding the D&I science workforce [22–24]. These efforts include workshops, seminars, and conferences both at the individual organizational level, as well as regional and national initiatives [25]. Best practices and the Teaching for Implementation Framework (TFI) have been put forth to provide guidance on how to train the translational workforce in D&I science [26]. The TFI focuses on differences in implementation *research* training and implementation *practice* training needed to build the D&I workforce [26]. To further support workforce development, efforts are underway within the NCATS existing Education Core Competencies Workgroup to build competencies that will enhance translational scientists’ knowledge and skills in D&I science [24].

Our findings align with the need to clarify the specific competencies for D&I investigators, as well as the recognition that competencies and needs differ by phase of translation research and the specific partner implementing the work. That is, not every researcher needs to become an expert D&I scholar, but instead can recognize the basic principles of D&I science and establish a cross-disciplinary approach [4]. Integration of D&I training and competency building through collaboration with existing training programs supported by the CTSA (e.g., Research Training (T series) and Career Development (K series) programs) can help frame learning objectives for trainees to gain a better understanding of and proficiency in D&I science that is tailored to the research setting and trainee type.

### Overcoming bandwidth challenges

Bandwidth challenges are prevalent across institutions focused on D&I initiatives, including at MUSC. Nearly half of CTSAs reported an inadequate D&I science workforce (45.7%) [22]. Specific challenges include that few faculty are formally trained in D&I, limiting the ability to provide appropriate training and consultations to those requesting these services; and there is no current guidance on the best way to staff D&I initiatives. As some examples, our program infrastructure includes four faculty with a small proportion of professional effort time dedicated to supporting DISC and one program manager; other D&I initiatives have reported three part-time faculty and three full-time academic staff members, and other sites reported a director, core faculty, and PhD level coordinator supported by a team of research assistants specializing in D&I science (e.g., PhD students and 1-2 masters students) [21]. Further assessment and guidance on the best approach for staffing D&I programs is needed.

Other approaches to overcome bandwidth challenges amidst the growing demand for D&I support is to encourage cross-CTSA collaborations. This includes sharing lessons learned or best approaches for delivering D&I services among leadership at institutions, sharing resources across hubs, and facilitating learning and networking outside of home institutions (e.g., participation in existing external training programs, webinars, and peer networks) [23].

Given that the needs currently exceed the capacity of D&I programs, creating resources and toolkits that provide answers and training for commonly asked questions continues to be critical. These initiatives could occur both locally, as well as nationally to help leverage existing resources and reduce redundancies [7]. Locally, toolkits could include topics that are of specific need to the community, as well as promotion of D&I innovations or interventions that have been developed by MUSC researchers. For example, the University of Wisconsin currently hosts 52 toolkits focused on providing D&I efforts within their institution. This type of toolkit could include checklists, training and promotional materials about the specific innovation, and evaluation tools for the implementation of the program [4,21]. Other effective resources include the use of decision aids to help investigators and D&I program staff determine whether a project is a D&I study and the types of additional services required [4]. Additionally, leveraging national toolkits and decision aids can help reduce local burden and bandwidth challenges. National toolkits have broadly focused on building skills that are foundational to D&I science, including: identifying and using theories, frameworks, and models; selecting measures; identifying and applying D&I strategies; selecting evaluation approaches; and dissemination of findings.

### Supporting full integration of D&I science within the CTSA

It is challenging to assess the overall impact of D&I-focused initiatives. Metrics reported by DISC include grant submissions, supported projects, consultations, and publications. While DISC has a robust system to track these initiatives, they do not fully capture the impact of the DISC initiative at our institution. Mehta (2021) suggests that a more comprehensive evaluation structure is needed to fully assess the impact of these initiatives within CTSA infrastructure and ensure D&I principles are truly integrated within the CTSA. Specifically, this includes distinguishing between products created by D&I scientists and the application of D&I methods, as well as tracking adoption, implementation, and scale-up of interventions into practice. Clarifying and standardizing the metrics of success could help ensure full understanding of the impact of D&I science initiatives and ensure the efforts are fully realized within the CTSA. This approach could also shift away from process evaluations that are most commonly used at sites and toward more robust impact evaluations [4].

Recommended approaches to fully integrating D&I science within the CTSA infrastructure include supporting pilot funding that requires the integration of D&I sciences into research projects, creating pilot awards that are specifically designed to advance the science of D&I, and/or funds to support the development of implementation packages that are public facing (e.g., manual for broad scale-up) [21]. Currently, pilot funding for D&I scientific research projects is provided directly or indirectly by 75.7% of CTSA programs [21].

A long-term strategy for integrating D&I within CTSAs and across the full translational continuum includes alignment with existing institutional initiatives. MUSC, like many other CTSA sites, is an academic health system. We therefore incorporate a learning health system approach to help ensure research fundings are informing clinical practice and that clinical practice is informing research via virtuous cycles. Given the emphasis on a learning health system approach, clarifying the ways that D&I science can contribute to the learning health system can help advance the role of D&I science within institutions and result in strengthened relationships between research and clinical practice [27].

## Conclusion

Since its 2019 inception, MUSC’s DISC has made significant strides in advancing research and practice. DISC addresses workforce development, offering training and mentorship to researchers and community partners, and tackles bandwidth challenges through cross-institutional collaborations and resource development. Integrating D&I science within the CTSA infrastructure necessitates ongoing evaluation, metric standardization, and strategic alignment with institutional initiatives. Key strategies have included workforce training to improve capacity and skills in D&I research, mentorship and collaboration with researchers, and advancement of D&I methods to accelerate clinical and translational research.

## References

[1] Bauer MS , Kirchner J. Implementation science: what is it and why should I care? Psychiatry Res. 2020;283:112376. 31036287 10.1016/j.psychres.2019.04.025

[2] Shelton RC , Lee M , Brotzman LE , Wolfenden L , Nathan N , Wainberg ML. What is dissemination and implementation science?: an introduction and opportunities to advance behavioral medicine and public health globally. Int J Behav Med. 2020;27(1):3–20. 32060805 10.1007/s12529-020-09848-x

[3] Buchanan GJR , Filiatreau LM , Moore JE. Organizing the dissemination and implementation field: who are we, what are we doing, and how should we do it? Implement Sci Commun. 2024;5(1):38. 38605425 10.1186/s43058-024-00572-1 PMC11007902

[4] Brownson RC , Proctor EK , Luke DA , et al. Building capacity for dissemination and implementation research: one university’s experience. Implement Sci. 2017;12(1):104. 28814328 10.1186/s13012-017-0634-4 PMC5559847

[5] Davis R , D’Lima D. Building capacity in dissemination and implementation science: a systematic review of the academic literature on teaching and training initiatives. Implement Sci. 2020;15(1):97. 33126909 10.1186/s13012-020-01051-6 PMC7597006

[6] Villalobos A , Blachman-Demner D , Percy-Laurry A , Belis D , Bhattacharya M. Community and partner engagement in dissemination and implementation research at the National Institutes of Health: an analysis of recently funded studies and opportunities to advance the field. Implement Sci Commun. 2023;4(1):77. doi: 10.1186/s43058-023-00462-y 37438834 PMC10339604

[7] Viglione C , Stadnick NA , Birenbaum B , et al. A systematic review of dissemination and implementation science capacity building programs around the globe. Implement Sci Commun. 2023;4(1):34. 36973832 10.1186/s43058-023-00405-7 PMC10041476

[8] Begg MD , Crumley G , Fair AM , et al. Approaches to preparing young scholars for careers in interdisciplinary team science. J Investig Med. 2014;62(1):14–25. 10.231/JIM.0000000000000021 PMC3970261 24169319

[9] Stevens ER , Shelley D , Boden-Albala B. Barriers to engagement in implementation science research: a national survey. Transl Behav Med. 2021;11(2):408–418. 31958137 10.1093/tbm/ibz193

[10] Tabak RG , Padek MM , Kerner JF , et al. Dissemination and implementation science training needs: insights from practitioners and researchers. Am J Prev Med. 2017;52(3 Suppl 3):S322–S329. 28215389 10.1016/j.amepre.2016.10.005 PMC5321656

[11] MacGeorge CA , King K , Andrews AL , et al. School nurse perception of asthma care in school-based telehealth. J Asthma. 2022;59(6):1248–1255. 33730979 10.1080/02770903.2021.1904978

[12] Johnson EE , MacGeorge C , King KL , et al. Facilitators and barriers to implementation of school-based telehealth asthma care: program champion perspectives. Acad Pediatr. 2021;21(7):1262–1272. 33940203 10.1016/j.acap.2021.04.025

[13] Johnson EE , MacGeorge C , Andrews A , et al. Nurse perspectives regarding implementation of an asthma monitoring mobile health application in the school setting. Telemed J E Health. 2021;27(8):955–962. 34152858 10.1089/tmj.2021.0100 PMC8432601

[14] Kirkland EB , Johnson E , Bays C , et al. Diabetes remote monitoring program implementation: a mixed methods analysis of delivery strategies, barriers and facilitators. Telemed Rep. 2023;4(1):30–43. 36950477 10.1089/tmr.2022.0038 PMC10027345

[15] Guille C , Johnson E , Douglas E , et al. A pilot study examining access to and satisfaction with maternal mental health and substance use disorder treatment via telemedicine. Telemed Rep. 2022;3(1):24–29. 35720443 10.1089/tmr.2021.0041 PMC8989094

[16] Sterba KR , Johnson EE , Douglas E , et al. Implementation of a women’s reproductive behavioral health telemedicine program: a qualitative study of barriers and facilitators in obstetric and pediatric clinics. BMC Pregnancy Childbirth. 2023;23(1):167. 36906564 10.1186/s12884-023-05463-2 PMC10007723

[17] Johnson EE , Sterba KR , Goodwin AJ , et al. Implementation of an academic-to-community hospital intensive care unit quality improvement program. Qualitative analysis of multilevel facilitators and barriers. Ann Am Thorac Soc. 2019;16(7):877–885. 30822096 10.1513/AnnalsATS.201810-735OC

[18] Sterba KR , Johnson EE , Nadig N , et al. Determinants of evidence-based practice uptake in rural intensive care units. A mixed methods study. Ann Am Thorac Soc. 2020;17(9):1104–1116. 32421348 10.1513/AnnalsATS.202002-170OC PMC7722472

[19] Allen CG , Hunt KJ , McMahon LL , et al. Using implementation science to evaluate a population-wide genomic screening program: Findings from the first 20,000 In Our DNA SC participants. Am J Hum Genet. 2024;111(3):433–444. doi: 10.1016/j.ajhg.2024.01.004. 38307026 PMC10940017

[20] Allen CG , Judge DP , Levin E , et al. A pragmatic implementation research study for in our DNA SC: a protocol to identify multi-level factors that support the implementation of a population-wide genomic screening initiative in diverse populations. Implement Sci Commun. 2022;3(1):48. 35484601 10.1186/s43058-022-00286-2 PMC9052691

[21] Quanbeck A , Mahoney J , Kies K , Judge K , Smith M. Building capacity for dissemination and implementation to maximize research impact in a CTSA: the university of wisconsin story. J Clin Transl Sci. 2020;4(3):209–215. 32695490 10.1017/cts.2020.3 PMC7348013

[22] Shelton RC , Dolor RJ , Tobin J , et al. Dissemination and implementation science resources, training, and scientific activities provided through CTSA programs nationally: opportunities to advance D&I research and training capacity. J Clin Transl Sci. 2022;6(1):e41. 35574154 10.1017/cts.2022.377 PMC9066314

[23] Dolor RJ , Proctor E , Stevens KR , Boone LR , Meissner P , Baldwin LM. Dissemination and implementation science activities across the clinical translational science award (CTSA) consortium: report from a survey of CTSA leaders. J Clin Transl Sci. 2019;4(3):188–194. 32695487 10.1017/cts.2019.422 PMC7348014

[24] Mehta TG , Mahoney J , Leppin AL , et al. Integrating dissemination and implementation sciences within clinical and translational science award programs to advance translational research: recommendations to national and local leaders. J Clin Transl Sci. 2021;5(1):e151. 34527291 10.1017/cts.2021.815 PMC8411263

[25] Stevens KR , de la Rosa E , Ferrer RL , et al. Bootstrapping implementation research training: a successful approach for academic health centers. J Clin Transl Sci. 2021;5(1):e168. 34733544 10.1017/cts.2021.827 PMC8532185

[26] Leppin AL , Baumann AA , Fernandez ME , et al. Teaching for implementation: a framework for building implementation research and practice capacity within the translational science workforce. J Clin Transl Sci. 2021;5(1):e147. 34527287 10.1017/cts.2021.809 PMC8411269

[27] Bennett NM , Orlando E , Meissner P. Linking dissemination and implementation science to learning health systems: opportunities for clinical and translational science award institutions. J Clin Transl Sci. 2020;4(3):176–179. 32695485 10.1017/cts.2020.15 PMC7348031

